# Rectal prolapse following short-course radiotherapy for rectal cancer: report of a case

**DOI:** 10.1093/jscr/rjaa529

**Published:** 2020-12-28

**Authors:** Fabio Nocera, Markus von Flüe, Daniel C Steinemann

**Affiliations:** Clarunis, Pelvic Floor Unit, University Center for Gastrointestinal and Liver Diseases, St. Clara Hospital and University Hospital Basel, Basel, Switzerland; Clarunis, Pelvic Floor Unit, University Center for Gastrointestinal and Liver Diseases, St. Clara Hospital and University Hospital Basel, Basel, Switzerland; Clarunis, Pelvic Floor Unit, University Center for Gastrointestinal and Liver Diseases, St. Clara Hospital and University Hospital Basel, Basel, Switzerland; Department of Surgery, University Hospital Basel, Basel, Switzerland

## Abstract

Palliative short-course radiotherapy may be considered as an alternative to abdominoperineal resection in elderly patients with advanced rectal cancer.

A 92-year-old woman was diagnosed with a rectal prolapse after short-course radiotherapy; 2 months before she was diagnosed with advanced lower rectal cancer. A curative approach was declined. Therefore, a palliative short-course radiotherapy followed. Two weeks after termination of radiotherapy, a symptomatic rectal prolapse has been observed. Endoscopy confirmed a tumor completely included in the prolapsing rectum. A rectal prolapse resection by Altemeier’s technique was performed. Histological examination downgraded the tumor staging to ypT1 M0.

This case discusses whether the prolapse was preexisting and led to overstaging the tumor or whether the prolapse is a new-onset complication of the radiotherapy.

It seems of paramount importance to detect preexisting rectal prolapse to avoid overstaging. If presumed rectal prolapse was not present before therapy, rectal prolapse may represent a new-onset adverse event of short-course radiotherapy.

## INTRODUCTION

Abdominoperineal resection for rectal cancer in frail patients is associated with a mortality up to 18% in short course and up to 51% within 1 year [1]. Palliative short-course radiotherapy represents an alternative to abdominoperineal rectal resection in frail patients with rectal cancer [2]. Combined with oxaliplatin-based chemotherapy a median survival of 11.5 months has been achieved. Subsequent surgery for colostomy formation may be avoided (80% of cases) [3]. Adverse events after short-course radiotherapy were observed in 72.3% of the cases [4].

In this case a symptomatic rectal prolapse was observed early after palliative short-course radiotherapy in an elderly woman. The question is whether the rectal prolapse was preexisting and led to overstaging of the tumor or if the prolapse was a sequela of the radiotherapy. This work has been written in accordance with the SCARE criteria [5].

## CASE PRESENTATION

**Figure 1:**
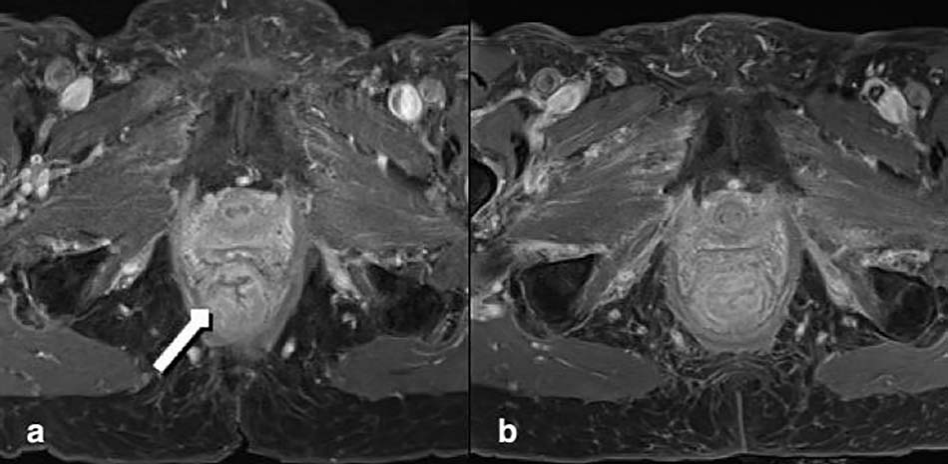
MRT Imaging little pelvis. (**a)** Before radiotherapy. Tumor appears in the lower rectum with suspicion of muscle infiltration (long arrow). (**b)** Imaging after short-course radiotherapy.

A 92-year-old woman, with known dementia and no previous history of anorectal surgery, was referred to the emergency department with a full-thickness rectal prolapse. Two months before she was diagnosed with advanced lower rectal cancer. Initially treated for hemorrhoids, a colonoscopy was arranged when patient noticed a resistance while introducing suppositories. Comorbidities were coronary heart disease and bronchial asthma. Family history was bland regarding tumor diseases. The colonoscopy demonstrated a malignant polyp of 4 cm size (3–4 cm above the anal verge). There was no clinical or endoscopic evidence of full-thickness rectal prolapse. The histology showed a moderately differentiated microsatellite-stable adenocarcinoma. A computed tomography and a magnetic resonance tomography (MRT) resulted in a cT3–T4 cN1 cM0, circumferential resection margin (CRM) positive (0 mm) staging (Fig. 1a). The multidisciplinary tumor board recommended an abdominoperineal resection. Despite detailed discussion with the patient and relatives a curative approach was declined, inter alia, because of age, comorbidities and dementia. Therefore, a palliative short-course radiotherapy with 5 × 5 Gy was performed [6].

Two weeks after the radiotherapy the patient noticed severe perianal pain. The patient’s relatives observed a painful partially reducible perineal bulge; thus, a rectal prolapse was diagnosed. At presentation in the emergency room the prolapsed segment of the rectum appeared edematous. The endoscopy showed a residual tumor completely included in the prolapsing rectum. MRT confirmed severe edema in the entire rectum after radiation and prolapse with poor definition of the tumor (Fig. 1b). The indication for perianal rectal prolapse resection was given and performed by Altemeier’s technique.

**Figure 2:**
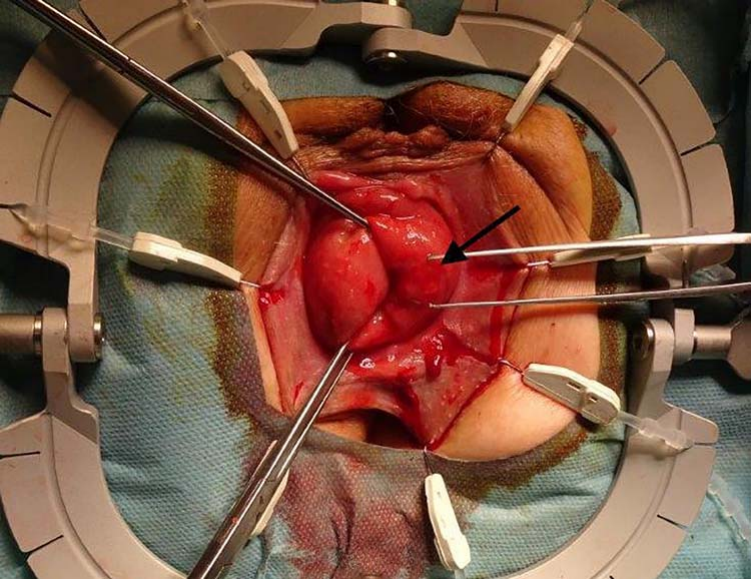
Adjusted surgical site using a Lonestar retractor. The rectal prolapse is exposed using Allis clamps. The tumor appears between three and six in lithotomy position (long arrow).

**Figure 3:**
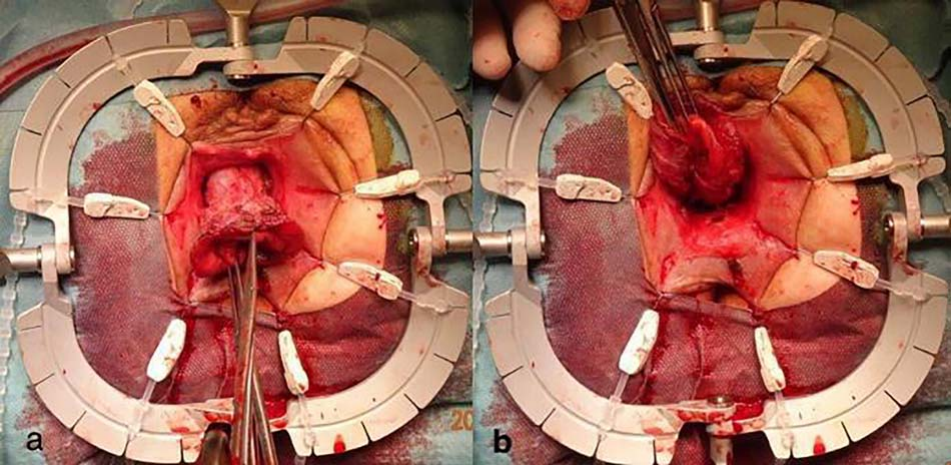
Incised rectal prolapse before circular dissection from the rectum. (**a)** Front view. (**b)** Rear view.

**Figure 4:**
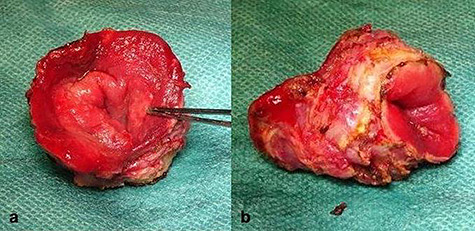
Completely resected tumor of rectum, 3.6-cm long and up to 3.0 cm in diameter. (**a)** Front view. (**b)** lateral view.

The surgical site was adjusted using a Lonestar retractor and the prolapse was exposed further using Allis clamps (Fig. 2). A monopolar current incision was made slightly above the dentate line, followed by circular dissection of the rectum starting from the ventral aspect (Fig. 3). The entire tumor area between three and six in lithotomy position (3 cm in diameter) was completely reduced (Fig. 4). No penetration was visible on the serosa side. The prolapse was then shortened and a tension-free coloanal anastomosis was created using several Monocryl 4.0 sutures.

The patient recovered uneventfully and was discharged on the 11th postoperative day. The postoperative course was delayed due to the patient’s fragility and initial mild pain problems. The histological examination confirmed a moderately differentiated, microsatellite-stable intestinal adenocarcinoma with complete excision and no infiltration. Thus, the TNM classification could be downgraded to ypT1 pN0 (0/1) L0 V1 Pn0 R0 M0 G2. In respect of the multimorbidity and the dementia of the patient no further oncological treatment was recommended.

## DISCUSSION

There are several reported cases of rectal adenocarcinoma detected in a prolapsed rectum [7–9]. No case of new-onset rectal prolapse after radiotherapy was described.

Incontinence and anal bleeding were noticed by the patient several months before the treatment and led to the diagnosis of rectal cancer. Although these symptoms are well explained by rectal cancer, they might also be associated with a rectal prolapse. Nevertheless, the patient’s relatives never noticed rectal prolapse before the radiotherapy.

Before treatment the tumor was staged cT3–T4 and an infiltration of the puborectal muscle was suspected. The tumor infiltration into the pelvic floor could have prevented the rectum from prolapsing. The response to the radiotherapy though was very distinct, as the tumor was staged ypT1 in the specimen. A tumor regression grade 2 according to Dworak was seen by the pathologist. The rate of complete pathological response 4–19 weeks after short-course radiotherapy was reported to range between 4.4% and 25% in the literature. A downstaging is achieved in 46.8% of cases [10]. The degree of downstaging only 2 weeks after termination of the radiotherapy is thus surprising.

On one hand, the influence of the rectal prolapse on the initial staging and on the other hand, promotion of the rectal prolapse by the radiotherapy, must be discussed.

If the rectal prolapse was present before the radiotherapy but not diagnosed, it may have contributed to an overstaging of the tumor in the MRT. Other cases of rectal prolapse associated with rectal or sigmoid cancer are reported [7–9]. Some authors postulated that sigmoid and rectal masses can form the lead point for intussusceptions, and consequently, patients present rectal prolapse more frequently [7]. It seems to be important to investigate the presence of rectal prolapse in newly diagnosed cancer, as this might influence the staging and treatment strategy.

If we presume rectal prolapse occurring after radiotherapy, then this is a novel adverse effect by the radiotherapy. The prolapse of the rectum could be fostered by the strong downgrading of the tumor, thus dissolving the tumor attachment to the pelvic floor.

Because of the acute presentation, the extensive edema that made it difficult to reduce the prolapse and the degree of suffering, a watchful waiting strategy was not possible. Perineal resection of the rectal prolapse according to Altemeier seems a safe and effective technique for this kind of situation.

This work presents a case of newly diagnosed rectal prolapse after short-course radiotherapy for advanced rectal cancer. It is of paramount importance to detect preexisting rectal prolapse to avoid overstaging. If rectal prolapse is not present prior to therapy, promotion of the rectal prolapse may occur by radiotherapy.
